# Development of a novel DNA sequencing method not only for hepatitis B virus genotyping but also for drug resistant mutation detection

**DOI:** 10.1186/1755-8794-6-S1-S15

**Published:** 2013-01-23

**Authors:** Fanjun Wang, Lili Lu, Changshun Yu, Zhanwu Lv, Xuelian Luo, Chao Wan, Zhaohui Hu, Qinyi Zhu, Youping Deng, Chuyu Zhang

**Affiliations:** 1 State Key Laboratory of Virology and College of Life Sciences, Wuhan University, Wuhan 430072, P.R. China; 2 Guangzhou Kingmed Center for Clinical Laboratory, Guangzhou 524035, P.R. China; 3 Wuhan University of Science and Technology, Wuhan 430081, P.R. China

## Abstract

**Background:**

In HBV-infected patients, different genotypes of the hepatitis B virus influence liver disease progression and response to antiviral therapy. Moreover, long-term antiviral therapy will eventually select for drug-resistant mutants. Detection of mutations associated to antiviral therapy and HBV genotyping are essential for monitoring treatment of chronic hepatitis B patients.

**Results:**

In this study, a simple method of partial-S gene sequencing using a common PCR amplification was established for genotyping clinical HBV isolates sensitively, which could detect the drug-resistant mutations successfully at the same time.

**Conclusions:**

The partial S gene sequencing assay developed in this study has potential for application in HBV genotyping and drug resistant mutation detection. It is simpler and more convenient than traditional S gene sequencing, but has nearly the same sensitivity and specificity when compared to S gene sequencing.

## Background

Eight distinct genotypes (A to H) of hepatitis B virus (HBV) have been identified, and this classification is based on the distance of the nucleotide sequence from the viral genome of 8% or greater [1,2]. These genotypes also have a distinct geographical distribution, while genotypes B and C are more common in China. Since genotypes of HBV influence liver disease progression and response to antiviral therapy in HBV-infected patients, several methods have been developed for genotyping of HBV strains [3], these include sequence analysis; microarray (DNA-Chip) [4,5]; reverse hybridization [6]; restriction fragment length polymorphism (RFLP) [7]; serological assays and genotype-specific PCR assays [8,9]. These techniques have the disadvantage that they are based on specific hybridization of HBV DNA, and nucleotide changes can interfere with this process and subsequent sequence analysis. For example, RFLP and multiple PCR methods might give wrong results even for a single base mutation. Serological assay has a low cost and does not rely on PCR amplification, but it is still subjected to the effects of specific base mutation. INNO-LiPA HBV genotyping assay has the limitation of high cost, and also its likelihood to be affected by the specific binding site mutant gene [10,14]. Sequence analysis is definitely the most accurate method and not subject to these constraints, but it is also the most labor intensive technique and needs nested PCR to increase the sensitivity.

As we all know, there are several antiviral therapies--such as interferon; pegylated interferon or nucleotide/nucleoside analogs--widely used to treat HBV infection. None of these therapies can eradicate HBV infection and all often induce drug-resistant mutants. As a result, HBV genotyping and the detection of mutations that confer drug resistance help select an appropriate treatment strategy and monitor the treatment. However, there are a limited number of methods that enable simultaneous genotyping and mutation detection. In this study, we used the partial S-gene sequencing using common PCR to genotype HBV, which is simpler and more sensitive compared with the S-gene sequencing.

Partial S-gene sequencing means sequencing part of the S gene from 370 nt to 861 nt. This part of the S gene we chose is relatively conserved and has many drug-resistant mutant sites, so it could be used in both HBV genotyping and in analysis of HBV drug resistant mutation.

## Results and discussion

### Phylogenetic tree analysis

**Figure 1:**
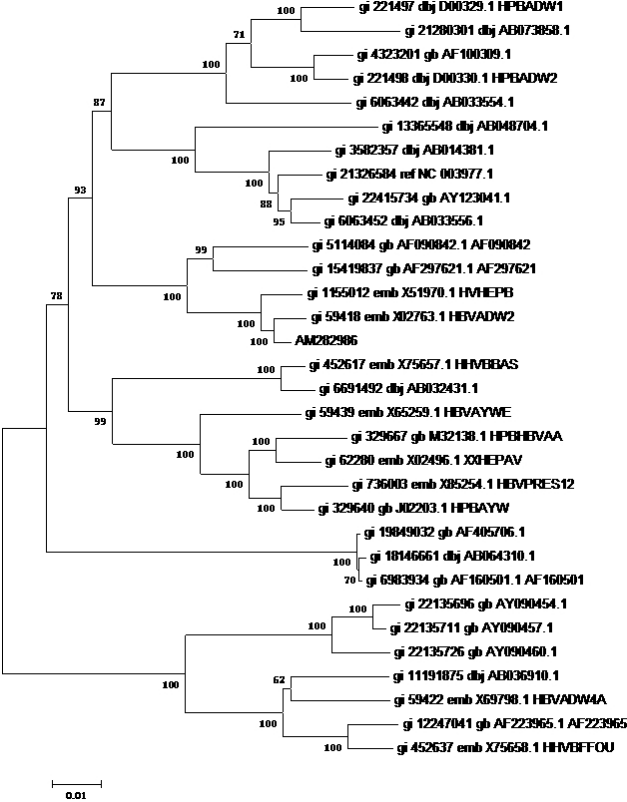
**Phylogenetic tree map of HBV genome sequencing**. 32 HBV genomes of eight different genotypes were sequenced by HBV genome sequencing, and software MEGA4 was used to analyze these genomes to get the phylogenetic trees of genome sequencing. **Tree Inference**: [*Method*: Neighbor-Joining; *Phylogeny Test and options*: Bootstrap (1000 replicates; seed = 100000)]; **Include Sites**: [*Gaps/Missing Data*: Complete Deletion]; **Substitution Model**: [*Model*: Nucleotide: Maximum Composite Likelihood; *Substitutions to Include*: d: Transitions + Transversions; *Pattern among Lineages*: Same (Homogeneous); *Rates among sites*: Uniform rates]

**Figure 2:**
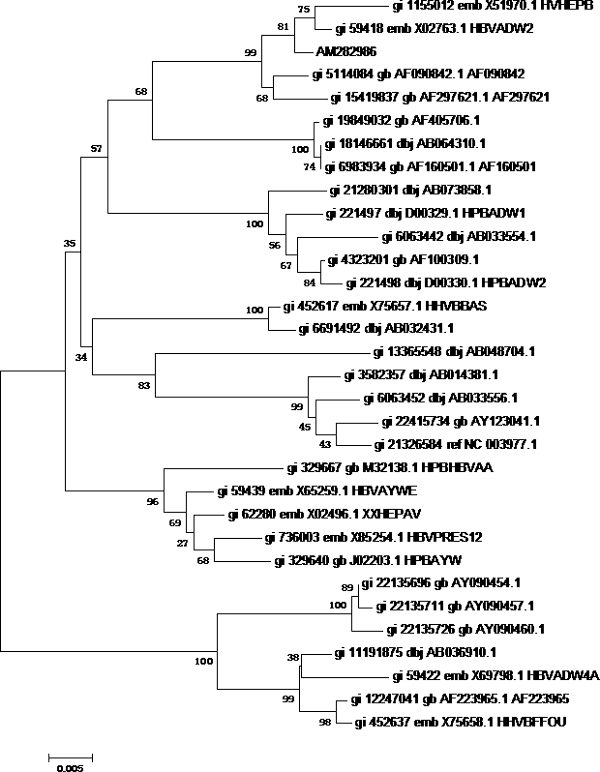
**Phylogenetic tree map of HBV S gene sequencing**. 32 HBV genomes of eight different genotypes were sequenced by HBV S gene sequencing, and software MEGA4 was used to analyze these genomes to get the phylogenetic trees of HBV S gene sequencing. **Tree Inference**: [*Method*: Neighbor-Joining; *Phylogeny Test and options*: Bootstrap (1000 replicates; seed = 100000)]; **Include Sites**: [*Gaps/Missing Data*: Complete Deletion; *Codon Positions*: 1st+2nd+3rd]; **Substitution Model**: [*Model*: Nucleotide: Maximum Composite Likelihood; *Substitutions to Include*: d: Transitions + Transversions; *Pattern among Lineages*: Same (Homogeneous); *Rates among sites*: Uniform rates]

**Figure 3:**
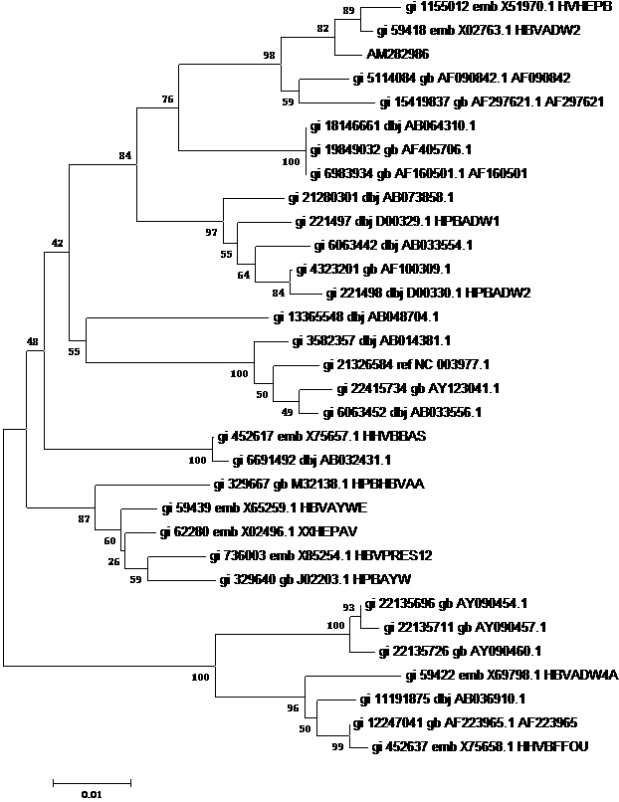
**Phylogenetic tree map of HBV partial S gene sequencing**. 32 HBV genomes of eight different genotypes were sequenced by HBV partial S gene sequencing, and software MEGA4 was used to analyze these genomes to get the phylogenetic trees of HBV partial S gene sequencing. **Tree Inference**: [*Method*: Neighbor-Joining; *Phylogeny Test and options*: Bootstrap (1000 replicates; seed = 100000)]; **Include Sites**: [*Gaps/Missing Data*: Complete Deletion; *Codon Positions*: 1st+2nd+3rd]; **Substitution Model**: [*Model*: Nucleotide: Maximum Composite Likelihood; *Substitutions to Include*: d: Transitions + Transversions; *Pattern among Lineages*: Same (Homogeneous); *Rates among sites*: Uniform rates]

First, phylogenetic tree analysis was used for testing the possibility of HBV genotyping using partial S gene sequencing. Reference sequences from 32 HBV genomes of eight different genotypes were used (shown as Table 1). Software MEGA4 was used to analyze these genomes to get the phylogenetic trees of genome sequencing; S gene sequencing and partial S gene sequencing respectively. All these sequences could be genotyped successfully by partial S gene sequencing. The results of phylogenetic tree analysis indicated that the partial S gene sequencing had nearly the same phylogenetic tree as that of the S gene sequencing (shown as Figure 1, 2, 3).

**Table 1:** Reference sequences for genotyping

Genotype A	AM282986	gi_59418	gi_1155012	gi_15419837	gi_5114084
Genotype B	gi_21280301	gi_221497	gi_221498	gi_4323201	gi_6063442
Genotype C	gi_13365548	gi_22415734	gi_6063452	NC_003977	gi_3582357
Genotype D	gi_329640	gi_736003	gi_329667	gi_62280	gi_59439
Genotype E	gi_452617	gi_6691492			
Genotype F	gi_11191875	gi_59422	gi_12247041	gi_452637	
Genotype G	gi_18146661	gi_6983934	gi_19849032		
Genotype H	gi_22135696	gi_22135711	gi_22135726		

**Figure 4:**
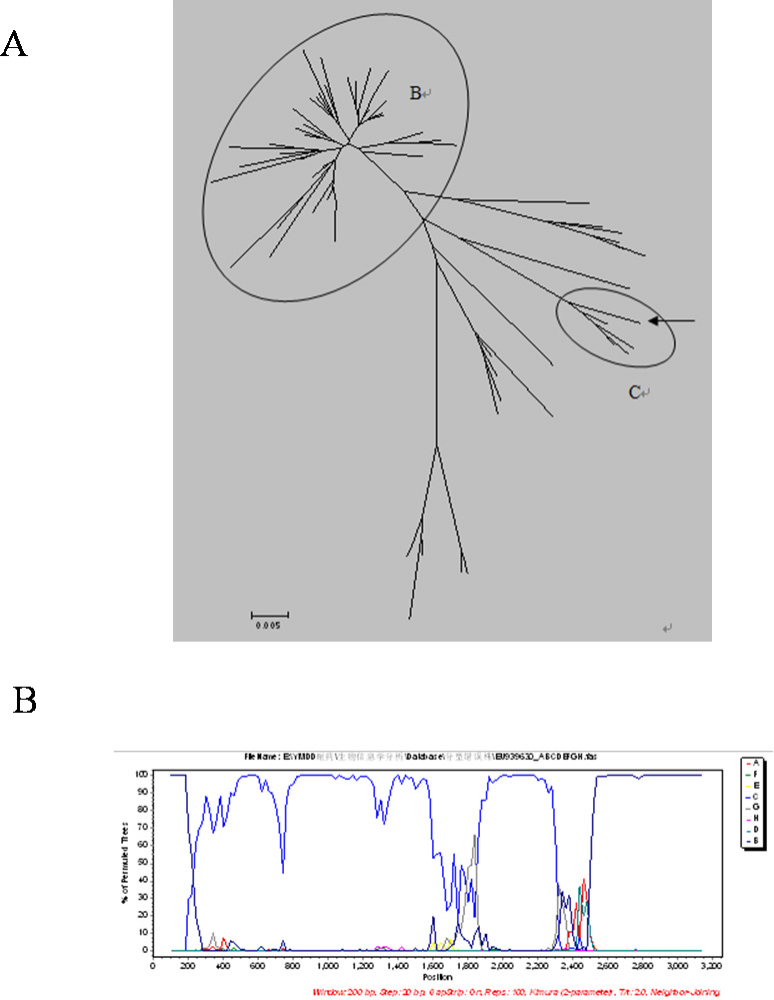
**Genotype and recombinant analysis results of EU939630**. A: Genotype results of 43 HBV genotype B samples using partial S gene sequencing, 42 samples of which had been genotyped correctly except one, EU939630. The arrow indicates EU939630 was genotyped as HBV genotype C by partial S gene sequencing. B: Recombinant analysis result of EU939630. From the recombinant analysis, we found that EU939630 was a C/B recombinant strain.

To further evaluate the effect of partial S gene sequencing, 53 HBV genotype A samples; 43 HBV genotype B samples; 50 HBV genotype C samples and 51 HBV genotype D samples were genotyped using partial S gene sequencing and S gene sequencing respectively (shown as Table 2). Compared the results of these two methods, we found that the results of partial S gene sequencing are consistent with S gene sequencing, except one sample, EU939630. From further recombinant analysis, we found that EU939630 was a C/B recombinant strain (shown as Figure 4).

**Table 2:** Sequences for genotyping verification

Genotype A	AB014370	AB330372	AB453986	AJ627227	AM295800	EU594388
	AB126580	AB330373	AB453987	AJ627228	AM410963	EU594389
	AB194950	AB453979	AB453988	AM184125	AM494718	EU594390
	AB194951	AB453980	AB453989	AM184126	AP007263	EU594391
	AB194952	AB453981	AJ309369	AM282986	EU594383	EU594392
	AB205118	AB453982	AJ309370	AM295795	EU594384	EU594393
	AB241114	AB453983	AJ309371	AM295797	EU594385	EU594394
	AB241115	AB453984	AJ344115	AM295798	EU594386	EU594395
	AB330371	AB453985	AJ627226	AM295799	EU594387	
Genotype B	AB014366	AB205119	AB287317	AB287326	EF473977	EU939628
	AB033554	AB205120	AB287318	AB287327	EU595030	EU939629
	AB033555	AB205121	AB287319	AB287328	EU595031	EU939630
	AB115551	AB205122	AB287320	AB287329	EU796066	
	AB117759	AB241117	AB287321	AB365445	EU796067	
	AB195933	AB287314	AB287322	AB368295	EU796068	
	AB195934	AB287315	AB287323	AJ627225	EU796071	
	AB195935	AB287316	AB287325	EF473976	EU939627	

Genotype C	AB014360	AB014374	AB014384	AB026811	AB033557	AB112065
	AB014362	AB014376	AB014385	AB026812	AB042282	AB112066
	AB014363	AB014377	AB014389	AB026813	AB042283	AB112348
	AB014364	AB014378	AB014391	AB026814	AB042284	AB112471
	AB014365	AB014379	AB014392	AB033550	AB042285	AB112472
	AB014367	AB014380	AB014393	AB033551	AB105172	
	AB014369	AB014381	AB014394	AB033552	AB105173	
	AB014371	AB014382	AB014396	AB033553	AB105174	
	AB014372	AB014383	AB014399	AB033556	AB112063	

Genotype D	AB033558	AB109475	AB119253	AB188244	AB210822	EU594431
	AB033559	AB109476	AB119254	AB188245	EU594422	EU594432
	AB090268	AB109477	AB119255	AB205126	EU594423	EU594433
	AB090269	AB109478	AB119256	AB205127	EU594424	EU594434
	AB090270	AB109479	AB120308	AB205128	EU594425	EU594435
	AB104709	AB110075	AB126581	AB210818	EU594426	EU594436
	AB104710	AB116266	AB188241	AB210819	EU594427	
	AB104711	AB119251	AB188242	AB210820	EU594428	
	AB104712	AB119252	AB188243	AB210821	EU594430	

### Sensitivity of partial S gene sequencing

147 HBV-positive (HBV copies were more than 500 copies/ml) serum samples were sequenced by partial S gene sequencing, 2 of which could not to be sequenced, so the sensitivity of our partial S gene sequencing was 98.64%.

### Application of partial S gene sequencing in recombinant HBV isolates

**Figure 5:**
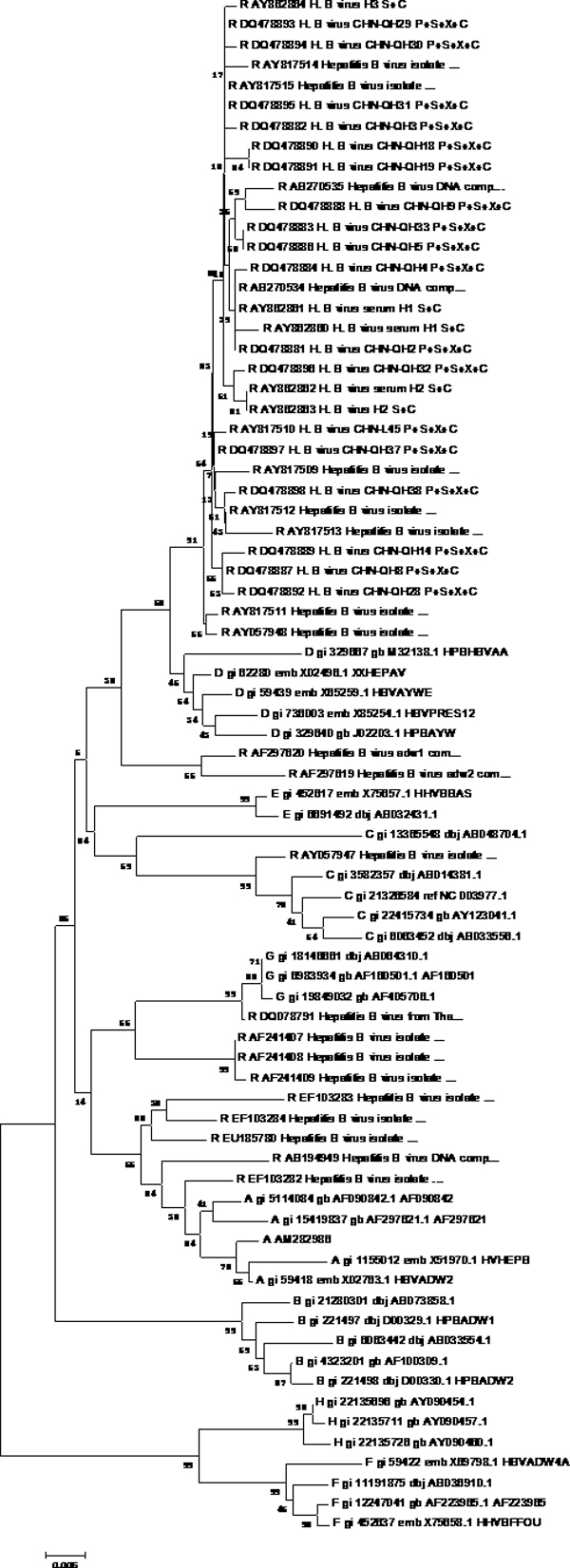
**Phylogenetic tree map of HBV recombinant strains using S gene sequencing**. 44 recombinant HBV isolates were collected to draw the phylogenetic tree map of HBV S gene sequencing by the method described above. **Tree Inference**: [*Method*: Neighbor-Joining; *Phylogeny Test and options*: Bootstrap (1000 replicates; seed = 100000)]; **Include Sites**: [*Gaps/Missing Data*: Complete Deletion]; **Substitution Model**: [*Model*: Nucleotide: Maximum Composite Likelihood; *Substitutions to Include*: d: Transitions + Transversions; *Pattern among Lineages*: Same (Homogeneous); *Rates among sites*: Uniform rates]

**Figure 6:**
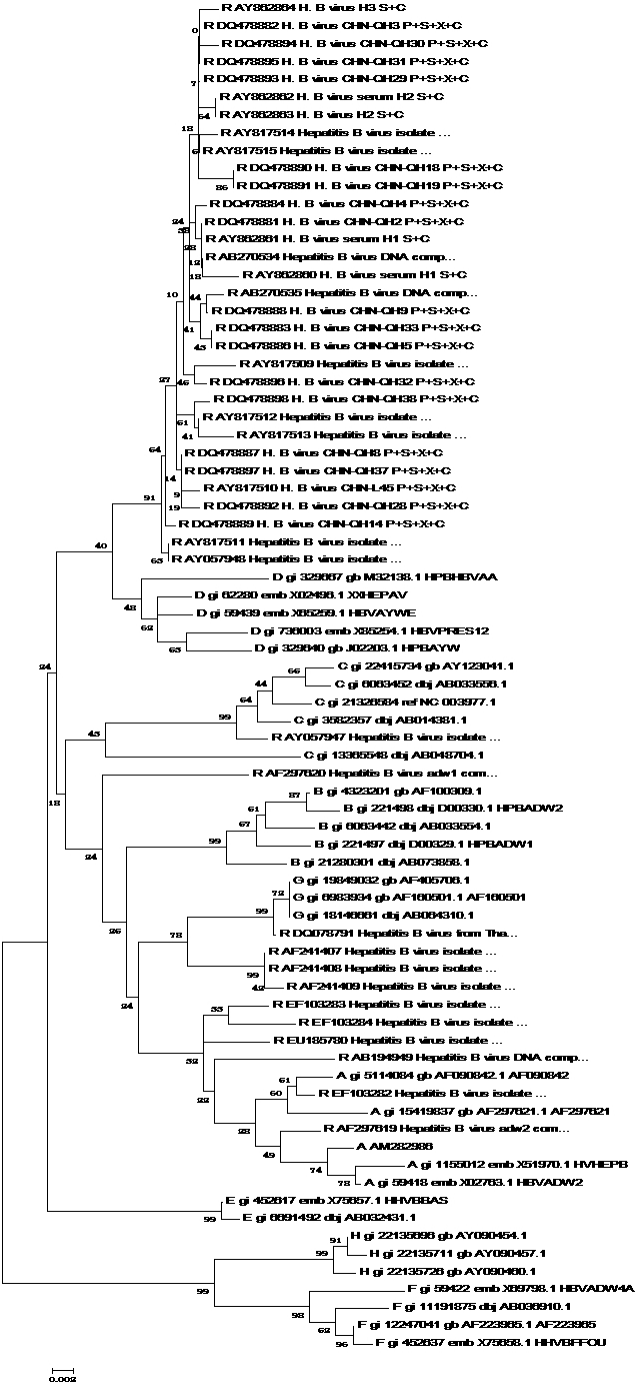
**Phylogenetic tree map of HBV recombinant strains using partial S gene sequencing**. 44 recombinant HBV isolates were collected to draw the phylogenetic tree map of HBV partial S gene sequencing by the method described above. **Tree Inference**: [*Method*: Neighbor-Joining; *Phylogeny Test and options*: Bootstrap (1000 replicates; seed = 100000)]; **Include Sites**: [*Gaps/Missing Data*: Complete Deletion]; **Substitution Model**: [*Model*: Nucleotide: Maximum Composite Likelihood; *Substitutions to Include*: d: Transitions + Transversions; *Pattern among Lineages*: Same (Homogeneous); *Rates among sites*: Uniform rates]

**Figure 7:**
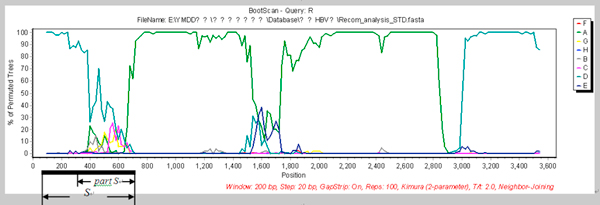
**Recombinant analysis results of AF297620**. AF297620 was failed to be genotyped by partial S gene sequencing because it had recombinant sites in partial S gene region.

Next, we evaluated the application of partial S gene sequencing in HBV recombinant isolates (shown as Figure 5 and 6). 44 recombinant HBV isolates were collected as shown in Table 3. The genotyping results indicated that there were 38 samples with the same results using partial S gene sequencing and S gene sequencing. Other 6 samples had different genotyping results by these two methods, which were isolated from South Africa (2/6); Thailand (1/6) and Vietnam (3/6) respectively. One sample from South Africa (AF297620) failed to genotype by partial S gene sequencing, as there was a recombinant site in its S region (shown as Figure 7) which was analyzed by Simplot software and NCBI Viral genotyping tool.

**Table 3:** Genotype results of 44 HBV recombinant strains

	Genotyping method			Source
		
	***S***	***partial S***	genome	
EU185780	A	A	D3/A2	Argentina
AB194949	A	A	A (A3/Acmr)/E	Cameroon
AY817509	D	D	C/D	China
AY817510	D	D	C/D	China
AY817511	D	D	C/D	China
AY817512	D	D	C/D	China
AY817513	D	D	C/D	China
AY817514	D	D	C/D	China
AY817515	D	D	C/D	China
AY862860	D	D	C/D	China
AY862861	D	D	C/D	China
AY862862	D	D	C/D	China
AY862863	D	D	C/D	China
AY862864	D	D	C/D	China
DQ478881	D	D	C/D	China
DQ478882	D	D	C/D	China
DQ478883	D	D	C/D	China
DQ478884	D	D	C/D	China
DQ478886	D	D	C/D	China
DQ478887	D	D	C/D	China
DQ478888	D	D	C/D	China
DQ478889	D	D	C/D	China
DQ478890	D	D	C/D	China
DQ478891	D	D	C/D	China
DQ478892	D	D	C/D	China
DQ478893	D	D	C/D	China
DQ478894	D	D	C/D	China
DQ478895	D	D	C/D	China
DQ478896	D	D	C/D	China
DQ478897	D	D	C/D	China
DQ478898	D	D	C/D	China
AY057947	C	C	A/C	China
AY057948	D	D	C/D	China
EF103282	A	A	A/D	India
EF103283	A	A	A/D	India
EF103284	A	A	A/D	India
AB270534	D	D	C/D	Mongolia:Ulaanbaatar
AB270535	D	D	C/D	Mongolia:Ulaanbaatar
AF297619	D	A	A/D	South Africa
AF297620	D	-	A/D	South Africa
DQ078791	C	G	G/C	Thailand
AF241407	D	G	C/A/G/B	Vietnam
AF241408	D	G	C/A/G/B	Vietnam
AF241409	D	G	C/A/G/B	Vietnam

### Drug-resistant mutations analysis

**Figure 8:**
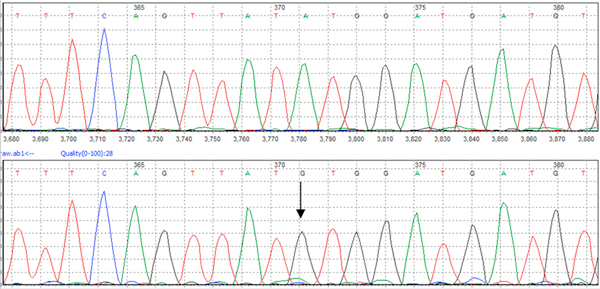
**Drug resistant mutation analysis by partial S gene sequencing**. Through drug resistant mutation analysis of 147 HBV positive serum samples using partial S gene sequencing, one sample was found to be rtM204V mutation. The arrow indicates the A is replaced by G.

The part of the S gene we chose for sequencing has many drug-resistant mutant sites, which means it had potential for use in analysis of HBV drug resistant mutation. To evaluate this, we analyzed all possible sites of resistance mutations (V521L; A529V; A529T; T532A; S550I; rtL180M; rtM204V/I; N584T and K589E) of 147 HBV-positive serum samples [16]. Through partial S gene sequencing we detected rtM204V mutation in one sample successfully (shown in Figure 8), meaning the partial S gene sequencing could be used in both HBV genotyping and in analysis of HBV drug resistant mutation.

## Conclusions

HBV genotyping and the detection of drug resistance mutations are important for monitoring the treatment of chronic hepatitis B, but there are a limited number of methods for the simultaneous detection of HBV genotypes and drug resistance mutations [11,13]. We have established a partial S gene sequencing method to genotype HBV isolates as well as detect drug resistance mutations at the same time. To testify the sensitivity of our partial S gene sequencing, 147 clinical serum samples were used, and 145 samples could be sequenced successfully by this assay, with a sensitivity of 98.64%.

The part of S gene we chose for sequencing has many drug-resistant mutant sites, and we detected all possible mutant sites in this region of 145 samples and found the rtM204V mutation in one sample. That means the partial S gene sequencing could be used in analysis of HBV drug resistant mutation.

To evaluate the potential of partial S gene sequencing for use in HBV genotyping, the phylogenetic tree analysis was used. From the phylogenetic tree mapping, we found that the partial S gene sequencing had nearly the same phylogenetic tree map to that of the S gene sequencing (shown as Figure 2 and 3). This means partial S gene sequencing has the possibility to be used as a promising method in HBV genotyping. To further demonstrate this, 197 HBV positive serum samples of four different genotypes (A, B, C, and D) were genotyped using partial S gene sequencing and S gene sequencing respectively (Table 2). Although at least eight HBV genotypes have been reported, the major HBV genotypes in China are B and C [12,15]. Genotypes A and D are found in a very small proportion of Chinese patients, and genotypes E, F, G, and H have not been reported in China. As a result, we only evaluated the performance of partial S gene sequencing assay for genotypes A, B, C, and D in our study. We found the results of partial S gene sequencing were consistent with S gene sequencing, except one sample, EU939630, which was proved to be a C/B recombinant strain. From this, we have proved that this assay could specifically detect mutant and wild-type HBV in clinical serum samples.

Considering that the recombinant strain might disturb genotyping effect, we evaluated the application of partial S gene sequencing in 44 HBV recombinant isolates (Table 3). 43 samples could be genotyped correctly by partial S gene sequencing method, meanwhile, S gene sequencing genotyped 41 samples successfully. That means partial S gene sequencing had the potential to take the place of S gene sequencing in the field of HBV genotyping.

Based on these findings, we could draw the conclusion that the partial S gene sequencing assay developed in this study could be applied in HBV genotyping and drug resistant mutation detection. It might be an ideal choice for HBV genotyping for it is simpler and more convenient than traditional S gene sequencing while it has nearly the same sensitivity and specificity as S gene sequencing.

## Methods

### Serum samples

Serum samples are collected from four hospitals: the Second People Hospital of Guangdong Province, the First Affiliated Hospital of Guangzhou Medical College, Guangzhou Overseas Chinese Hospital, and Guangzhou Huadu Ren-Ai Hospital. All these serum samples were collected in compliance with the Helsinki Declaration, and all the patients who provided serum samples were voluntary. This study was approved by the Institutional Review Board of Wuhan University. All specimens were sampled from sterile blood vessels (excluding anticoagulant) and stored at -20°C.

### Sequencing

HBV DNA was isolated from serum (QIAamp DNA Blood Mini Kit, QIAGEN, Hilden, Germany) according to the kit instructions. A product of 491 base pairs of partial S gene was amplified with the primers F (sense, 5'- TCGCTGGATGTGTCTGCGGCGTTTTAT-3') and R (antisense, 5'- ACCCCATCTTTTTGTTTTGTTAGG-3') using a PCR protocol as follows: 12 min at 95°C, 35 cycles of 1 min at 94°C, 1 min at 52°C, 1 min at 72°C, and a final elongation step of 7 min at 72°C, using the AmpliTaq Gold amplification system.

### Phylogenetic tree analysis

To testify the feasibility of the partial S gene sequencing used for HBV genotyping, 32 different genotypes of the HBV (http://lancelot.otago.ac.nz) genome sequence were chosen as the reference sequences (shown in Table 1). Three different DNA sequencing methods were used to genotype serum samples for phylogenetic tree mapping: whole genome sequencing, S gene sequencing and partial S gene sequencing.

## Competing interests

The authors declare that they have no competing interests.

## Authors' contributions

Fanjun Wang designed this study; Lili Lu and Youping Deng drafted and revised the manuscript; Changshun Yu, Zhanwu Lv participated in the collection of clinical samples and gene sequencing; Xuelian Luo, Chao Wan participated in the phylogenetic tree analysis; Zhaohui Hu, Qinyi Zhu participated in acquisition of data and data analysis; Youping Deng gave final approval of the version to be published; Chuyu Zhang participated in the design of the study and the general supervision of the research group.
